# Recent Advances in Liver Transplantation for Hepatocellular Carcinoma

**DOI:** 10.1007/s11864-024-01247-8

**Published:** 2024-08-01

**Authors:** P. Jonathan Li, Sachin Shah, Neil Mehta

**Affiliations:** 1 University of California San Francisco School of Medicine, 533 Parnassus Avenue, San Francisco, CA 94143 USA; 2 Department of Medicine, University of California San Francisco, San Francisco, CA USA; 3 Division of Gastroenterology, Department of Medicine, University of California San Francisco, San Francisco, CA USA

**Keywords:** Hepatocellular carcinoma, Immunotherapy, Downstaging, Machine perfusion, Liver transplantation, Biomarkers

## Abstract

Liver transplantation for hepatocellular carcinoma (HCC) remains an evolving field. Major challenges HCC transplant patients face today include liver organ donor shortages and the need for both better pre-transplant bridging/downstaging therapies and post-transplant HCC recurrence treatment options. The advent of immunotherapy and the demonstrated efficacy of immune checkpoint inhibitors in multiple solid tumors including advanced/unresectable HCC hold promise in expanding both the neoadjuvant and adjuvant HCC transplant treatment regimen, though caution is needed with these immune modulating agents leading up to and following transplant. New options for pre-transplant HCC management will expand access to this curative option as well as ensure patients have adequate control of their HCC prior to transplant to maximize the utility of a liver donor. Machine perfusion has been an active area of investigation in recent years and could expand the organ donor pool, helping address current liver donor shortages. Finally, additional HCC biomarkers such as AFP-L3 and DCP have shown promise in improving risk stratification of HCC patients. Together, these three recent advancements will likely alter HCC transplant guidelines in the coming years.

## Introduction

Liver cancer is a significant public health burden worldwide: it ranks fourth in cancer related deaths and is projected to cause > 1 million deaths each year by 2030 [1]. Hepatocellular carcinoma (HCC) accounts for the majority (~ 90%) of primary liver cancer cases [2]. Liver transplantation is the only curative treatment for patients with early stage, unresectable HCC [3]. While liver transplantation was first attempted for HCC as early as the 1960’s, it wasn’t until Mazzaferro et al.’s landmark study in 1996 proposing the Milan Criteria that outcomes dramatically improved [4]. The Milan Criteria remains the basis for HCC liver transplant candidate selection in the US, but there have been notable additions to the Milan Criteria in US HCC transplant policy. For example, expansion criteria have been proposed as the Milan Criteria is now considered too restrictive [5, 6]. Additionally, alpha fetoprotein’s (AFP) prognostic value in HCC outcomes has led to multiple risk stratification tools and guided pre-transplant HCC management, HCC transplant candidate selection, and post-transplant surveillance [7–9].

Today, US HCC patients qualify for liver transplant if they meet Milan Criteria or are able to be successfully downstaged, with some initial tumor burden restraints, into Milan Criteria via locoregional therapies or neoadjuvant systemic therapies. Prioritization of all liver transplant candidates is based on MELD-Na score, but HCC liver transplant candidates meeting or successfully downstaged into the Milan Criteria qualify for MELD exception points after 6 months on the waitlist. Currently, MELD exception points are calculated based on the MMAT-3 system, and those with an AFP > 1000 ng/mL need to demonstrate a sustained drop in AFP levels to < 500 ng/mL to qualify for MELD exception points.

Despite current detailed practice guidelines, the management of HCC remains a dynamic and actively evolving area. Here, we aim to review recent developments in transplant for HCC, from pre-transplant treatment options and transplant candidate selection to post-transplant surveillance and adjuvant therapy options.

## Pre-Transplant HCC Management

### Downstaging Options in HCC

Liver transplant is the preferred treatment option for patients with HCC who fall within the Milan criteria because it removes both tumor(s) and underlying liver disease [10]. There has been an effort to expand selection criteria based on tumor size, number, and biology to offer liver transplant to more HCC patients. Downstaging is an option for patients to reduce tumor burden typically using local–regional therapy (LRT) to meet criteria for liver transplant [11]. Common options for downstaging have included transarterial chemoembolization (TACE), ablation either radiofrequency (RFA) or microwave (MWA), transarterial radioembolization (TARE), and stereotactic body radiotherapy (SBRT) [12]. Downstaging carefully selected patients with HCC has been associated with excellent post-transplant outcomes [13]. For example, one recent study evaluating 209 consecutive downstaged (UNOS-DS Criteria) HCC transplant patients from 7 transplant centers spanning 4 UNOS regions reported a post-transplant 2-year 95% survival rate and 7.9% recurrence rate [14].

The use of systemic treatment options as targeted therapies has also now extended beyond those with just advanced HCC. In 2007, the oral multikinase inhibitor sorafenib was proven to extend overall survival (OS) and used as first-line treatment in HCC. Lenvatinib, another oral multikinase inhibitor that targets vascular endothelial growth factor (VEGF) receptors, was later found to be non-inferior to sorafenib in overall survival in untreated advanced HCC in a phase 3 trial while cabozantinib, regorafenib, and VEGFR2-targeted monoclonal antibody, ramucirumab, have been effective as second-line therapy [10, 15]. Immune checkpoint inhibitor (ICI) immunotherapy is an exciting advancement in the treatment of solid tumors including HCC. The next few sections will focus on immunotherapy in HCC, its use in the neoadjuvant setting, its use in combination with LRT as well as ongoing clinical trials looking at its use in downstaging and/or bridging prior to liver transplantation.

### Immunotherapy in Advanced HCC

Immunotherapy has played a pivotal role in the treatment of individuals with advanced HCC. Nivolumab, a PD1 ICI, was one of the first to be studied for the treatment of advanced HCC. The Checkmate 040 was an open-label, noncomparative, dose escalation and expansion trial that included 262 patients (48 in the dose-escalation phase and 214 in the dose-expansion phase) and found an objective response rate (ORR) of 20% in the dose-expansion phase and 15% in dose-escalation phase, which was considered durable ORR and demonstrated nivolumab’s utility as a treatment option [16]. Pembrolizumab is another PD1 ICI that was assessed in the KEYNOTE 224 trial for treatment of HCC. This was a non-randomized, multicenter, open-label, phase 2 trial that included 104 patients with an ORR of 17% in those who had previously been treated with sorafenib [17]. In an additional study on pembrolizumab, the KEYNOTE-394 trial, a randomized, double-blind, phase 3 study found that pembrolizumab significantly improved OS, progression-free survival (PFS), and ORR compared to placebo in a primarily Asian population [18]. Similarly, tislelizumab. an anti-PD-1 ICI and was studied in an open-label, multicenter, phase 3 randomized control trial and was noninferior for OS compared to sorafenib as first-line treatment [19].

The IMbrave150 trial was a hallmark trial in the management of HCC. This was an open-label phase III randomized control trial that compared the PDL1 ICI atezolizumab and VEGF inhibitor bevacizumab with sorafenib in the treatment of advanced HCC. This study totaled 501 patients (336 assigned to receive atezolizumab plus bevacizumab and 165 assigned to receive sorafenib) with median OS significantly higher at 19.2 months in the atezolizumab plus bevacizumab group compared to 13.4 months with sorafenib (HR 0.66; 95% CI 0.52–0.85; p < 0.001). Furthermore, the median PFS was significantly higher in the atezolizumab plus bevacizumab group at 6.9 months compared to 4.3 months in the sorafenib group. This led to atezolizumab plus bevacizumab becoming first-line treatment in advanced HCC [20, 21].

Other forms of combination therapy have also been effective in the management of patients with advanced HCC. The use of tremelimumab (anti-CTLA-4) in combination with durvalumab (anti-PDL1) was studied in an open-label, phase 3 trial that investigated OS in patients randomly assigned to receive tremelimumab plus durvalumab or durvalumab or sorafenib in those with unresectable HCC. The combination of durvalumab and tremelimumab had significantly higher median OS of 16.4 months compared to sorafenib 13.8 months (survival HR 0.78 96.02% CI, 0.65 to 0.93; p = 0.0035) [22].

### Neoadjuvant use of Immunotherapy in HCC

With immunotherapy’s growing role in advanced HCC management, researchers have examined whether immunotherapy could improve outcomes in the neoadjuvant setting in those with resectable disease. In one single arm phase 1B study, the use of neoadjuvant cabozantinib and nivolumab was examined in patients with HCC who did not meet traditional resection criteria. The study included 15 patients, of which 12 had negative margins on resection and 5 of those 12 had major pathologic responses [23]. In another single center, open-label, randomized phase 2 trial that investigated neoadjuvant nivolumab compared to nivolumab plus ipilimumab (anti-CTLA-4 monoclonal antibody) for one dose prior to surgery in patients with resectable HCC. Of the 27 patients enrolled in the study, 7 had surgical cancellations but importantly not related to treatment related adverse events. Median PFS was 9.4 months with nivolumab group and 19.5 months in the nivolumab plus ipilimumab group. Of the 20 patients who underwent resection, 3 of the 9 patients in the nivolumab group had major pathologic response compared with 3 of 11 patients in the nivolumab plus ipilimumab group [24]. These studies provide promising evidence for potential use of ICI in the neoadjuvant setting.

### Combination Immunotherapy with LRT

The use of ablative therapies in combination with ICI is based on the premise that ablative therapies can induce a peripheral immune response and therefore enhance the effect of ICI. TACE remains one of the most widely used LRT in patients with intermediate stage HCC. EMERALD-1 is a randomized, double-blind, placebo-controlled, multicenter phase 3 trial investigating the efficacy and safety of durvalumab when given with either DEB-TACE or TACE followed by durvalumab with or without bevacizumab in patients with locoregional HCC. Initial results have demonstrated durvalumab in combination with TACE and bevacizumab had a statistically significant and clinically meaningful improvement in their primary endpoint (PFS) with the clinical trial still ongoing examining secondary endpoints [25, 26]. There are several other ongoing phase 2 and 3 clinical trials investigating LRT with TACE and systemic ICI therapy in intermediate stage HCC [11].

Other trials looking at different forms of LRT in combination with ICI are also being investigated. The ROWAN study is a global, open-label, prospective, multicenter randomized control trial looking at the possibility of TARE with yttrium-90 (Y-90) glass microspheres followed by durvalumab in combination with tremelimumab compared to TARE alone in HCC. The primary objective is assessing ORR and durability of local tumor control with estimated timeline of completion of the study December 2027 [27].

### Use of Immunotherapy in Downstaging/Bridging HCC Prior to Liver Transplantation

An emerging ICI application is in downstaging and/or bridging therapy to increase the number of patients eligible for liver transplantation. Certainly, there are safety considerations to weigh to prevent liver transplant graft rejection as well as other associated adverse events.

One of the first clinical trials, PLENTY202001, (NCT04425226) is an unblinded randomized control trial that is investigating the use of pembrolizumab and lenvatinib as a downstaging and/or bridging therapy prior to liver transplant in patients with HCC. The primary outcome is RFS and ORR and secondary outcomes include disease control rate, percentage of participants who experience adverse events, and percentage of participants who discontinue treatment due to adverse events. Results of this study are expected in December 2024 [28]. Table 1 summarizes several other ongoing clinical trials using ICI as a method of downstaging and/or bridging HCC prior to liver transplantation.

**Table 1:** Ongoing clinical trials examining systemic immunotherapy as a method of downstaging and/or bridging prior to liver transplant

NCT/Trial ID	Systemic ICI Therapy Arms	Design	Primary Endpoint
NCT05027425	Durvalumab and Tremelimumab	Single-arm, phase II	Post-transplant rejection
NCT05475613	Anti-PD-1 inhibitor (tislelizumab, pembrolizumab, nivolumab et al.)	Single-arm, phase II	2-year relapse-free survival rate
NCT04425226/PLENTY 202001	Pembrolizumab plus lenvatinib	Randomized, parallel assignment	RFS, ORR
NCT04035876	Camrelizumab plus apatinib	Single-arm phase I/II	RFS, ORR

### Biomarkers for Liver Transplantion Candidate Selection

AFP is the primary biomarker used for liver transplant candidate selection today. Despite AFP’s strong prognostic value, additional biomarkers for HCC liver transplant candidate selection are needed. Most HCC patients on the liver transplant waitlist today are well below the AFP threshold for MELD exception points and have low to normal AFP levels by the time they receive a liver transplant. However, post-liver transplant HCC recurrence still occurs in 10–15% of patients. Given that treatment options for post-LT recurrences are limited and survival after recurrence is poor, selection of candidates who would derive the most benefit from liver transplantation, particularly given the current organ donor shortage, becomes even more critical today. As the pre-transplant treatment arsenal expands with emerging combination locoregional therapy and immunotherapy/systemic therapy options, additional metrics that assess treatment response in greater resolution and better predict important HCC outcomes (i.e. post-liver transplantation HCC recurrence and tumor progression on the liver transplant waitlist) will help improve risk assessment and liver transplant candidate selection.

AFP-L3 and des gamma carboxyprothrombin (DCP) are emerging biomarkers that could supplement AFP in risk stratifying HCC patients being considered for liver transplant. The role of AFP-L3 and DCP is well established in the context of HCC surveillance and diagnosis. For example, the GALAD score, which incorporates age, gender, AFP, AFP-L3, and DCP to assess probability of HCC in patients with chronic liver disease, has been validated in multiple patient populations [29–31]. However, the evidence for AFP-L3 and DCP’s value in predicting other HCC transplant outcomes, particularly in the US patient population, has been limited until recent years.

A recent prospective single center study demonstrated the prognostic value of AFP-L3 and DCP in predicting high risk explant pathology [32]. This study included 153 patients and defined high risk explant pathology as presence of microvascular invasion, poor tumor differentiation, and/or viable tumor burden beyond Milan Criteria. On multivariable logistic regression, elevated AFP-L3 (≥ 15%) and DCP (≥ 7.5 ng/mL) were associated with high-risk explant pathology whereas elevated AFP (≥ 100 ng/mL) was not. The findings of this study were expanded upon in a follow up single center prospective study comprising of 285 patients that evaluated the ability of AFP-L3 and DCP to predict post-liver transplant HCC recurrence [33]. There, patients with elevated AFP-L3 (≥ 15%) and DCP (≥ 7.5 ng/mL) had a 3-year recurrence free survival of 43.7% compared to 97% in patients with normal AFP-L3 and DCP. Given these findings, AFP-L3 and DCP may also play a role in liver transplant candidate selection since patients with high likelihood of recurrence may not derive the most benefit from liver transplant and better identify aggressive tumor biology that results in cancer progression despite LRT or other neoadjuvant therapies while on the transplant waitlist.

Currently, all HCC patients who qualify for MELD Exception receive equal priority on the transplant waitlist. However, there remains a spectrum of HCC tumor biology and prognosis among those who qualify for liver transplantation with MELD Exception. Studies have demonstrated the range of waitlist dropout probability due to tumor progression and that increased likelihood of waitlist dropout correlates with worse post-liver transplant outcomes. For example, one study utilized UNOS data from before the 6-month MELD exception points delay policy was implemented to develop a waitlist dropout risk score using patients from long waitlist regions and validate the risk score using patients from short and medium waitlist regions [34]. Among patients who made it to liver transplantation in the short and medium waitlist regions, the study also demonstrated that higher waitlist dropout risk was associated with worse post-liver transplant recurrence free survival. The association of waitlist dropout risk with post-liver transplant outcomes supports the idea to prioritize HCC liver transplant candidates and/or tailor HCC pre-transplant management based on waitlist dropout risk. A follow up study using machine learning models to develop a more accurate waitlist dropout risk score was completed, but the performance of the final machine learning model was similar likely given the absence of any novel prediction variables utilized for model development [35].

More recently, AFP-L3 and DCP have also been shown to be important for predicting waitlist dropout in a prospective single center study [36]. Among the study’s 267 HCC patients listed for liver transplantation, those with elevated AFP-L3 (≥ 35%) or elevated DCP (≥ 7.5 ng/mL) had 60% chance of waitlist dropout due to tumor progression, clinical deterioration, or death within 2 years of listing and those with both biomarkers elevated had a 100% chance of waitlist dropout within the same time period. Notably, AFP was not a significant predictor of waitlist dropout in univariable and multivariable cox proportional hazards regression analysis. Thus, AFP-L3 and DCP appear to play important roles in HCC prognosis in the pre-transplant setting, especially in an era where limited range of AFP levels in most HCC patients undergoing liver transplant is beginning to diminish AFP’s prognostic value. Select studies examining AFP-L3 and DCP’s prognostic value in HCC outcomes are summarized in Table 2.

**Table 2:** Recent AFP-L3 and DCP Studies

Primary Outcome	Score/Biomarker Cutoffs	Performance/Results	Clinical Application
Post-LT Time to HCC Recurrence in LDLT Patients [45]	MoRAL score = 11 × √PIVKA + 2 × √AFP	Concordance Index = 0.84 (95% CI: 0.76–0.91)	LDLT HCC Transplant Candidate Selection
High Risk Explant Pathology [32]	AFP-L3 at LT ≥ 15% DCP at LT ≥ 7.5 ng/mL	Multivariate Odds Ratio for High Risk Explant: - Elevated AFP-L3 4.47** (95% CI 1.45 – 13.73) - Elevated DCP 9.30*** (95% CI 2.97 – 29.11)	Transplant Candidate Selection + Post-LT Risk Stratification
Post-LT Time to HCC Recurrence [33]	AFP-L3 at LT ≥ 15% DCP at LT ≥ 7.5 ng/mL	3-year Post-LT RFS: - Elevated AFP-L3 & DCP 43.7% (95% CI 20.3–65.2) - Normal AFP-L3 and DCP 97.0% (93.8–98.6)	Transplant Candidate Selection + Post-LT Risk Stratification
Post-Listing Time to Waitlist Dropout (Tumor Progression, Clinical Deterioration, Death) [36]	AFP-L3 at LT ≥ 35% DCP at LT ≥ 7.5 ng/mL	Multivariate Hazards Ratio for Time to WL Dropout: - Elevated AFP-L3 2.25* (95% CI 1.04 – 4.88) - Elevated DCP 2.20* (95% CI 1.15 – 4.20)	Pre-Transplant HCC Management, Transplant Candidate Selection, ECD Organ Allocation

p-value: * < 0.05 ** < 0.01 *** < 0.001

## Expanding Options for Liver Transplantation

Liver transplant for both HCC patients and patients with end stage liver disease is currently limited by a shortage of organ donors. Moreover, one study projected that the aging population and increasing prevalence of obesity and diabetes will continue to negatively affect donor liver quality, increase discard rates, and possibly decrease access to liver transplantation in the coming decade [37]. Use of extended criteria donors, living donors, and machine perfusion techniques could expand access to liver donors.

### Extended Criteria Donors for HCC Recipients

Extended criteria donors generally include donors with advanced age, significant liver steatosis and/or transmissible infections (i.e. Hepatitis B/C, HIV, etc.) as well as donation after circulatory death donors (DCD). The primary concern with extended criteria donors is with allograft function after transplantation but inflammatory sequelae from adverse events such as ischemia–reperfusion injury have been hypothesized to impact oncologic outcomes as well. One study utilized the UNOS database to compare recurrence free survival and overall survival after liver transplantation among HCC patients transplanted with MELD exception between 2012 and 2016 [38]. While the study noted no overall difference in recurrence free survival between patients who received a donor after brain death (DBD) donor and patients who received a DCD donor, subset analysis of patients at higher risk of post-LT recurrence based on RETREAT score favored DBD donors. It is unclear why patients at increased risk of post liver transplantation recurrence, without identified recurrence, have worse survival with DCD donors than DBD donors. Recurrence reporting is not mandated by UNOS, so underestimation of recurrence rates could have had an impact on the results of the study. However, two cited previous single center studies also observed no difference in recurrence free survival between DBD and DCD donors. Although the association warrants further exploration, the observed difference in a large national database could inform donor allocation policies for HCC liver transplant candidates specifically. For example, those with less HCC tumor burden and/or well-treated lesions pre-transplant may opt to wait longer for a DBD donors as the risks associated with DCD donors at this time may not outweigh the risk of tumor progression.

### Living Donor Liver Transplantion for HCC

Living donor liver transplant (LDLT) remains a viable option for HCC transplant patients. While historically more utilized in Asian countries, LDLT volume has increased in the US in recent years for both end stage liver disease and HCC with up trending graft survival rates [39]. Previous studies observed increased post liver transplantation recurrence rates with LDLT, possibly attributed to the shorter waitlist time [40, 41]. It has been shown that both prolonged waitlist time and shortened waitlist time (< 6 months) is associated with slightly worse post liver transplantation outcomes and current policies specify a 6 month waiting period before MELD exception points are awarded for HCC patients awaiting DDLT [7, 42]. Notably, a more recent analysis of patients who were transplanted between 1998 and 2018 using the UNOS dataset did not identify a difference in recurrence rate between LDLT and DDLT [43]. Given the increasing shortage of liver donors today, LDLT remains a good alternative for carefully selected HCC patients who may be at high risk of waitlist dropout or who may be beyond conventional transplant criteria. However, selection criteria, such as those identified by Bhangui et al. (AFP < 100 ng/mL, within UCSF expanded criteria, PET Avid HCC), or risk threshold defined by scores such as the MoRAL Score (11 × √PIVKA + 2 × √AFP) proposed by Lee et al. are still needed to optimize utility of a LDLT [44, 45].

### Machine Perfusion

Normothermic and hypothermic machine perfusion for liver transplant is currently an active area of investigation with numerous ongoing clinical trials. The potential for machine perfusion to not only reduce post-transplant complications but also expand the liver donor pools comes at an opportune time [46–48]. For example, a recent trial comparing Hypothermic Perfusion to Static Cold Storage in DCD liver transplant recipients demonstrated a reduction in non-anastomotic biliary stricture as well as early allograft dysfunction [48]. Given the increased risk of ischemic cholangiopathy and early allograft dysfunction associated with DCD donors, the ability to reduce these complications via machine perfusion could have implications for expanding liver transplant access. Additionally, machine perfusion is thought to modulate the early immune response following liver transplantation by decreasing oxidative stress and subsequent inflammation. These impacts on the immune system have unique implications for HCC patients and post-transplant oncologic outcomes.

One recent European retrospective study examined the impact of hypothermic oxygenated liver perfusion (HOPE) on post-liver transplant HCC recurrence in DCD liver donor recipients [49]. The study was conducted at two centers and designed as follows: at the first center, post-liver transplant HCC recurrence rate was compared between HOPE DCD liver donor recipients and propensity score matched non-perfused DBD recipients whereas the second center compared recurrence rates between non-perfused DCD liver donor recipients and propensity score matched non-perfused DBD recipients. There was a significant difference in 5-year recurrence free survival (92% vs 73%; p = 0.027) between the HOPE DCD liver donor recipients and non-perfused DBD recipients at the first center whereas no difference was observed at the second center. The results demonstrate a benefit of HOPE DCD over non-perfused DBD liver donor recipients and suggest a benefit of HOPE DCD over non-perfused DCD. A follow up prospective study or randomized controlled trial with a larger patient population is certainly warranted to further investigate the benefits of HOPE DCD and other machine perfusion techniques, such as normothermic machine perfusion, in HCC patients.

## Post-Liver Transplantation Management

### Post-Transplant Recurrence Risk Prediction/Scores

Numerous post-transplantation recurrence risk prediction scores have been proposed in the last decade. One widely used recurrence risk score is the RETREAT (Risk Estimation of Tumor Recurrence After Transplant) Score, which was originally developed and validated in a multicenter North American patient cohort and has since been subsequently validated in the UNOS dataset and multiple European cohorts [9, 50–52]. The RETREAT Score awards points based on AFP at time of transplant, presence of microvascular invasion on explant pathology, and explant tumor burden, and its accuracy and simplicity has resulted in wide adoption. With the recognition of inflammatory indices as a potential prognostic variable for HCC outcomes, a few additional risk scores have incorporated neutrophil–lymphocyte-ratio (NLR) in addition to the variables included in RETREAT. For example, the MORAL (Model of Recurrence After Liver Transplantation) includes NLR, maximum pre-transplant AFP, largest pre-transplant tumor size, vascular invasion on explant, tumor grade on explant, and tumor size and number on explant [53]. While the MORAL score demonstrated strong discrimination, the development of the score from single center patient data spanning the course of over a decade during which numerous UNOS policy changes have occurred puts into question generalizability and applicability to HCC patients undergoing transplantation today, and external validation of the MORAL score is pending. More recently, the RELAPSE Score was developed using the US Multicenter HCC Transplant Consortium dataset and validated on an external European cohort [54]. The RELAPSE Score factors in pre-transplant NLR, pre-transplant maximum AFP, micro- and macro-vascular invasion on explant pathology, maximum tumor diameter on explant, and explant tumor grade, and demonstrated good discrimination (0.75 – 0.77) upon external validation.

Machine learning may further improve recurrence prediction models. To date, only a few studies have examined the utility of machine learning algorithms in predicting post-liver transplantation. One single center study evaluated multiple machine learning algorithms (CoxNet, Random Survival Forest, Support Vector Machines, and DeepSurv) and found that a model based on CoxNet performed best (C-Index 0.75) [55]. Importantly, their CoxNet machine learning model outperformed existing scores including the MORAL score. Further evaluation of the role of machine learning in improving HCC risk prediction models is warranted, but it is likely that the combination of machine learning models with more predictive biomarkers such as AFP-L3 and DCP will provide the next significant improvement in prediction model performance.

Despite the development and availability of numerous prediction models that perform reasonably well, there currently lacks a consensus risk stratification tool and, subsequently, a standardized post-liver transplantation recurrence surveillance protocol and adjuvant therapy guideline [7, 56]. Further validation and cost–benefit analysis of risk-score based surveillance or adjuvant therapy protocols is needed to realize the clinical utility of the currently available risk stratification tools.

### Adjuvant Therapy/Recurrence Management

Post-transplant HCC recurrence portends poor prognosis, and immunosuppression is one area researchers have considered optimizing to decrease risk of recurrence. Calcineurin inhibitors are common after liver transplant but have been hypothesized to promote tumor growth. The SiLVER-trial sought to determine whether the mammalian target of rapamycin (mTOR) inhibitor sirolimus improved post-liver transplant RFS and OS. While there was no difference in RFS or OS identified at the study endpoint, there was an improvement in RFS and OS observed in the first 3 and 5 years of post-liver transplantation follow up, respectively, and a subsequent analysis suggested that patients with pre-liver transplant AFP ≥ 10 ng/mL may derive a significant survival benefit from sirolimus [57, 58]. Thus, those with elevated AFP prior to liver transplant or at high risk of post-liver transplant recurrence based on risk scores such as RETREAT may benefit from mTOR based immunosuppression.

Post-liver transplant HCC recurrence remains challenging to manage. While prognosis is generally unfavorable, certain HCC recurrence features can indicate better post-liver transplant recurrence survival. For example, one study identified elevated AFP levels at the time of recurrence (≥ 100 ng/mL), early recurrence (within 1 year post-liver transplant), presence of microvascular invasion on explant pathology, and not being amenable to curative surgical or locoregional therapies portends worse survival after recurrence [59]. Adjuvant therapy may improve post-liver transplant survival in this subset of high-risk of patients, but there is no current guideline for selecting patients who would benefit from adjuvant therapy. Sorafenib has been the mainstay for post-liver transplant HCC recurrence treatment much of this past decade and has been proposed as adjuvant therapy in patients at high risk of post-liver transplant recurrence [60]. The efficacy of ICI in other cancers has sparked studies evaluating the role of immunotherapy as a pre-transplant bridging and/or down-staging systemic therapy. While recent trials discussed above have demonstrated increased efficacy of immunotherapy compared to sorafenib in patients with advanced/unresectable HCC, the results cannot be directly extrapolated to the liver transplant population, and the evidence supporting use of ICI for post-transplantation adjuvant therapy or recurrence management remains limited to case series at this time, though trials are pending [61]. Overall, the potential role of ICI as adjuvant therapy remains an active area of investigation given the balance that must be struck between suppressing allograft rejection and reinvigorating the tumor immune response. More precise manipulation of the immune system, through dendritic cell therapy and vaccines for example, could allow this balance to be better struck in the future [62, 63].

## Conclusions

The recent advancements in immunotherapy, biomarkers, and machine perfusion will continue to push the field of HCC liver transplantation care to evolve and adapt. While the evidence for the role of immunotherapy in HCC liver transplant patients remains to be firmly established, trials demonstrating their efficacy in advanced HCC patients and case reports highlighting the safety and efficacy of immunotherapy as bridging and post-liver transplantation HCC recurrence therapies show promise. Machine perfusion represents a tool that could safely expand the liver donor pool for HCC patients without compromising oncologic outcomes. Finally, the strength of AFP-L3 and DCP in predicting multiple HCC outcomes in studies thus far could eventually lead to updated guidelines for HCC liver transplant candidate selection, allocation of extended criteria liver donors, and post-transplant surveillance and adjuvant therapy protocols.

## Key References

- Imfinzi plus bevacizumab met primary endpoint for progression-free survival in liver cancer eligible for embolisation in EMERALD-1 Phase III trial. 2023. https://www.astrazeneca.com/media-centre/press-releases/2023/imfinzi-combination-improves-pfs-in-liver-cancer.html. Accessed 6 Feb 2024.
  - This reference is of major importance as it reports the primary endpoint of a Phase III randomized control trial and shows efficacy of combined ICI + Bevacizumab + TACE for HCC over TACE alone in earlier stage HCC. Once published, the full findings of this trial may have important implications for HCC transplant patients as TACE is a common LRT used in the pre-transplant for downstaging/bridging therapy.

- Norman JS, Li PJ, Kotwani P, Shui AM, Yao F, Mehta N (2023) AFP-L3 and DCP strongly predict early hepatocellular carcinoma recurrence after liver transplantation. J Hepatol 79:1469–1477.
  - This reference is of importance because it shows in a prospective cohort that the additional HCC biomarkers AFP-L3 and DCP significantly improve post-transplant HCC recurrence risk stratification.

- Mehta N, Kotwani P, Norman J, Shui A, Li P-Y, Saxena V, Chan W, Yao FY AFP-L3 and DCP are superior to AFP in predicting waitlist dropout in HCC patients: Results of a prospective study. Liver Transplantation 10.1097/LVT.0000000000000149.
  - This reference is of importance because it shows in a prospective cohort that the additional HCC biomarkers AFP-L3 and DCP may play an important role in identifying HCC patients on the liver transplant waitlist at high risk of experiencing tumor progression and dropping out.

- Silverstein J, Roll G, Dodge JL, Grab JD, Yao FY, Mehta N (2020) Donation After Circulatory Death Is Associated With Similar Posttransplant Survival in All but the Highest-Risk Hepatocellular Carcinoma Patients. Liver Transpl 26:1100–1111.
  - This reference is of importance because it shows in a large, US patient cohort that donation after circulatory death for HCC patients may be a viable alternative.

- Mueller M, Kalisvaart M, O‘Rourke J, et al (2020) Hypothermic Oxygenated Liver Perfusion (HOPE) Prevents Tumor Recurrence in Liver Transplantation From Donation After Circulatory Death. Annals of Surgery 272:759.
  - This reference is of importance because it demonstrates that machine perfusion may make donation after circulatory death donors a strong alternative for HCC patients without compromising oncologic outcomes.

## Data Availability

No datasets were generated or analysed during the current study.
